# COVID-19 Immunopathology and the Central Nervous System: Implication for Multiple Sclerosis and Other Autoimmune Diseases with Associated Demyelination

**DOI:** 10.3390/brainsci10060345

**Published:** 2020-06-04

**Authors:** Marina Kleopatra Boziki, Alexios-Fotios A. Mentis, Maria Shumilina, Gleb Makshakov, Evgeniy Evdoshenko, Nikolaos Grigoriadis

**Affiliations:** 1 2nd Neurological University Department, AHEPA General Hospital, Aristotle University of Thessaloniki, 54634 Thessaloniki, Greece; bozikim@auth.gr; 2 Public Health Laboratories, Hellenic Pasteur Institute, 11521 Athens, Greece; mentisaf@gmail.com; 3 Laboratory of Microbiology, University Hospital of Larissa, School of Medicine, University of Thessaly, 41110 Larissa, Greece; 4 SPb Multiple Sclerosis Centre, Dinamo pr 11, St. Petersburg 197110, Russia; m.shumilina@centrems.com (M.S.); g.makshakov@centrems.com (G.M.); e.evdoshenko@centrems.com (E.E.)

**Keywords:** SARS-CoV-2, COVID-19, multiple sclerosis, disease-modifying treatment, systemic lupus erythematosus, antiphospholipid syndrome

## Abstract

In the frame of the coronavirus disease 2019 (COVID-19) pandemic, recent reports on SARS-CoV-2 potential neuroinvasion placed neurologists on increased alertness in order to assess early neurological manifestations and their potentially prognostic value for the COVID-19 disease. Moreover, the management of chronic neurological diseases, such as Multiple Sclerosis (MS), underwent guided modifications, such as an Extended Interval Dose (EID) of Disease-Modifying Treatment (DMT) administration, in order to minimize patients’ exposure to the health system, thus reducing the risk of SARS-CoV-2 infection. In this review, we summarize existing evidence of key immune pathways that the SARS-CoV-2 modifies during COVID-19 and the relevant implication for MS and other autoimmune diseases with associated demyelination (such as Systemic lupus erythematosus and Antiphospholipid syndrome), including the context of potential neuroinvasion by SARS-Cov-2 and the alterations that DMT induces to the immune system. Moreover we hereby aim to provide an overview of the possible consequences that COVID-19 may carry for the Central Nervous System (CNS) in People with MS (PwMS) and other demyelinating diseases, which are likely to pose challenges for treating Neurologists with respect to the long-term disease management of these diseases.

## 1. Introduction

Over the last few months, the coronavirus disease 2019 (COVID-19), due to severe acute respiratory syndrome coronavirus 2 (SARS-CoV-2) infection, is posing a challenge to global health, and has dramatically changed the world health service landscape. Previously, other coronaviruses were known to cause severe respiratory disease in humans and were linked with epidemic outbreaks, such as the severe acute respiratory syndrome coronavirus (SARS-CoV) and the Middle East respiratory syndrome coronavirus (MERS-CoV), both leading to increased risk of mortality [1]. Endemic distribution of other coronaviruses has previously been reported, but the symptoms of the associated infection appear to be mild in general [2]. COVID-19 appears with symptoms similar to the previously known SARS and MERS diseases, the most common being fever, fatigue, dry cough, headache, nasal congestion, sore throat, myalgia, and arthralgia [3,4]. Nausea, vomiting, and diarrhea are not rare, but are more prominent in specific populations, such as in children. In rare instances, more specifically in elderly and/or immunocompromised individuals, fever may be absent, and this observation underlines the necessity of increased alertness to evaluate non-typical symptoms and to lower the threshold of SARS-CoV-2 status evaluation for physicians.

A proportion of severely affected patients will eventually develop acute respiratory distress syndrome (ARDS) and will require mechanical ventilation [5], with high mortality. Advanced age and concomitant comorbidities, such as hypertension, diabetes and cardiovascular disease are factors predisposing towards poor prognosis [5]. Under these circumstances, patients may show evidence of decreased mental status, thus rendering neurological evaluation at the peak of the disease challenging [6].

Although most of COVID-19 patients exhibit normal leukocyte count, leucopenia [5,7] and lymphocytopenia appear as a major laboratory finding in the severely affected patients [5]. Other markers of inflammation, such as C-reactive protein and erythrocyte sedimentation rate are also elevated, whereas increased concentration of interleukin (IL)-6 seems to characterize the cytokine storms associated with increased mortality [5]. Laboratory confirmation of COVID-19 is conducted by the establishment of the viral ribonucleic acid (RNA) presence by molecular techniques in nasal and pharyngeal swab, sputum, blood, feces and/or urine [8].

Currently, there is no specific antiviral treatment universally prescribed for COVID-19. Based on previous evidence stemming from the MERS and SARS epidemics, most of the COVID-19 patients are prescribed antiviral treatment [3,7]. Several antiviral agents, such as lopinavir/ritonavir and remdesivir, are currently in use but their efficacy remains to be verified by large real-world studies. The use of corticosteroids is being reserved for patients whose respiratory function rapidly deteriorates and/or show evidence of immune system excessive activation, as in the case of ARDS [9].

## 2. Neurological Manifestations of COVID-19

Since the outbreak of the pandemic, neurological symptoms have been described in patients with COVID-19. These neurological manifestations have posed significant challenge for neurologists worldwide, with respect to case management and treating decisions [10,11,12]. Most cases exhibit symptoms associated with the underlying pathology and may be observed in the context of hypoxia, thus posing a challenge for the physicians in terms of differential diagnosis. Such symptoms include headache, dizziness, mental state alterations, ataxia, convulsions and stroke-like episodes [13]. A case of SARS-CoV-2-associated meningitis, evidenced by SARS-CoV-2 RNA detected in the cerebrospinal fluid (CSF) and manifested with convulsion and unconsciousness, has recently been described [14]. Moreover, an apparent case of COVID-19-associated acute necrotizing encephalopathy (ANE) has been described [15]. This rare hemorrhagic encephalopathy has been previously reported as a consequence of other viral infections, such as influenza. Interestingly, ANE occurs in the absence of Central Nervous System (CNS) viral invasion and/or immune-mediated demyelination, though in the presence of the systemic cytokine storm effect and Blood–Brain Barrier (BBB) compromise [16]. Taken together, these cases pose a significant question as to whether COVID-19-associated meningitis and/or encephalopathy requires neuroinvasion on behalf of the virus or it may be a consequence of systemic cytokine disequilibrium.

The potential of SARS-CoV-2 to trigger immune-mediated neurological disease, at least with respect to the peripheral nervous system, has been elucidated by the reference of five SARS-CoV-2-associated cases with Guillain–Barré syndrome [17]. This report is consistent with previous observations of other coronavirus infections [18,19] that have been reported to lead in Guillain–Barré syndrome. Of note, SARS-CoV-2-associated Guillain–Barré syndrome needs to be distinguished from critical illness neuro-muscular disease, the latter being manifested following prolonged stay in the intensive care unit (ICU) and/or corticosteroid treatment. Loss of smell (“anosmia”) early in the disease course further elucidates early potential involvement of the peripheral nerves, in this case, namely, the olfactory nerve, in the frame of COVID-19. As a presenting manifestation of COVID-19, loss of smell has recently underlined the necessity for early neurological evaluation; however, the prognostic value of this finding remains to be verified. More importantly, this finding provides evidence of a potential route of entry of the SARS-CoV-2 in the CNS, carrying implication for long-term consequences with respect to neuroinflammation and/or neurodegeneration. As the loss of olfactory function is considered an early manifestation of neurodegenerative diseases, mainly in, but not restricted to, Parkinson’s disease [20], it is of special clinical and prognostic value to explore the link between early loss of smell and long-term alterations in non-motor symptoms following COVID-19, that may indicate an underlying neurodegenerative processes. Neurodegeneration is present also at early stages of Multiple Sclerosis (MS) and the assessment of cognition is suggested as a relevant clinical outcome of prognostic value for MS. Moreover, cognitive function, a previously underappreciated parameter, compared to the motor symptoms, has been known to be affected during MS relapses [21,22]. In the context of potential neuroinvasion by SARS-CoV-2 and its currently unknown latency in the CNS, we here suggest that the long-term assessment of cognition and overall neurological competence in patients that overcome the acute phase of COVID-19 may be of particular significance.

### 2.1. SARS-CoV-2 Receptors and Neuroinvasive Potential

Similar to SARS-CoV, the SARS-CoV-2 receptor-binding domain recognizes a host receptor with great similarity to the human angiotensin-converting enzyme 2 (ACE2), and the affinity of this recognition was shown to determine susceptibility towards SARS-CoV-2 infection [23,24,25,26].

In the renin–angiotensin–aldosterone system (RAAS), ACE2 degrades angiotensin II to angiotensin, thus overall inhibiting the RAAS and ameliorating vasoconstriction, sodium retention, and fibrosis. ACE2 is expressed by the lung alveolar epithelial cells, thus rendering the lung as the primary target organ for SARS-CoV-2 [24,27]. The vascular endothelium is also known to express the ACE2 receptors. It is thus possible that the BBB, via its vascular compartment, also provides a gate for viral invasion of the CNS. In this respect, the first report regarding the presence of SARS-CoV-2 in the CSF of the COVID-19 patient with meningitis provides direct evidence for the neuroinvasive potential of the virus [28]. As previous studies have indicated for other coronaviruses, such as the SARS-CoV and MERS-CoV, coronaviruses may invade the CNS [29,30,31,32,33] and, in this process, two distinct routes may be implicated, i.e., hematogenous spread or neuronal retrograde dissemination. The latter route may, at least in part, explain the early-phase loss of smell and taste in COVID-19-affected patients. Retrograde axonal transport has been demonstrated for infectious agents, including coronaviruses, through cranial nerves, such as the olfactory, the trigeminal, the glossopharyngeal, and the vagus nerves [32,34,35]. Furthermore, coronaviruses were previously shown to migrate from mechanoreceptors and chemoreceptors in the lung towards the brainstem and the associated cardiorespiratory center, via a synapse-connected route [14]. In an experimental setting, the direct invasion of the brainstem by coronavirus led to increased mortality due to respiratory dysfunction [36]. The loss of neurons in the CNS in the frame of coronavirus infection is likely to be attributed to neurodegeneration in the absence of inflammation [34]. The expression of ACE2 receptors has been described in neurons and glial cells, thus rendering these cell types susceptible to coronavirus [35]. In the event that SARS-CoV-2 exhibits a similar potential for trans-synaptic migration, across the olfactory nerve, towards the brainstem, this pathway may be accounted for by the loss of spontaneous respiratory function in the frame of severe COVID-19. Taken together, further clinical evidence is needed to elucidate whether early defects in olfaction, within the frame of COVID-19, may provide prognostic value with respect to the respiratory distress and the associated mortality.

### 2.2. Immune Response in COVID-19 and the Implications for Neurological Disorders

SARS-CoV-2 infection has been linked to immune system dysfunction manifested as lymphopenia in patients with COVID-19, and the proportion of lymphopenic patients is especially increased among severely affected individuals [37]. Moreover, alterations in the relative frequency of several immune cell subsets has been reported, such as the reduction of CD4+ T cells, CD8+ T cells, B cells, and natural killer (NK) cells, an effect more prominent also in patients with severe COVID-19. In particular, CD8+ T-cell relative frequency seems to be negatively correlated with markers of inflammation and sustained CD8+ T-cell reduction, even post-treatment, has been associated with poor prognosis [9].

Contrary to the total lymphocyte and the CD4+ and CD8+ T-cell subset reduction, patients with COVID-19 exhibit increased CD4+ T-cell relative frequency of the C-C motif chemokine receptor 6 (CCR6) positive T helper (Th) 17 effector phenotype [9], thus exhibiting over-activation of the immune system towards inflammation. This is in accordance with other pro-inflammatory cytokines that were reportedly increased in the peripheral blood of patients with COVID-19, such as the Th-17 inducing cytokine IL-6, IL-10, IL-2 and the Th1 signature cytokines interferon gamma (IFN)-γ and tumor necrosis factor (TNF)-α [38,39].

Cytokine storms are characterized by the overproduction of IL-7, IL-10, granulocyte-colony stimulating factor, (IFN)-γ induced protein 10, monocyte chemoattractant protein-1, macrophage inflammatory protein 1 alpha, TNF-α, and other pro-inflammatory mediators. They have been described in patients with COVID-19 that are severely affected in an ICU, and they have been linked with poor prognosis [40]. COVID-19-related cytokine storms closely resemble the cytokine release syndrome (CRS), a systemic inflammatory response, often following infection and/or drug toxicity, especially in the frame of immune therapies for cancer and immunosuppression [41,42]. IL-6 is a key mediator in CRS, as it promotes T cell expansion, more specifically Th17 lineage commitment, and B cell activation [43]. Similar to CRS, the cytokine storms observed in severe COVID-19 lead to ARDS and multiple organ failure [44]. Consistent with the assumption of an over-active immune system mediating a detrimental effect towards organ failure, abundant lymphocytic inflammatory infiltrates in the interstitial space of the lungs have been reported in patients with COVID-19-associated ARDS [9].

In this respect, tocilizumab, a recombinant humanized monoclonal antibody that blocks the IL-6 receptor, is an emerging treatment for COVID-19 currently on trial [45]. Tocilizumab is currently prescribed for patients with rheumatoid arthritis; however, by exhibiting the capacity to attenuate systemic inflammatory response, this immune-modifying agent serves as a candidate treatment against COVID-19 cytokine storms [46].

## 3. COVID-19 and MS, Comorbidities that Affect MS Prognosis, and Other Systemic Autoimmune Diseases with a Potential for Demyelination

People with MS (PwMS) exhibit an increased risk of infections compared to the general population. In the context of MS, infections have been shown to lead to significant morbidity and to contribute to disease exacerbation in the form of relapses and/or the worsening of neurological manifestations [47].

Coronavirus infection has been previously reported in patients with MS [48] and it may have played a role in triggering the disease, as with most environmental factors in genetically predisposed individuals [49]. This triggering effect may be linked to the virus’ capacity to initiate the responses of the innate and the adaptive immune system, as manifested by the SARS-CoV-2-associated alterations in immune cell populations and the respective T-cell, B-cell, and NK-cell subsets. Thankfully, recent preliminary data provide evidence that the majority of SARS-CoV-2-infected PwMS, also under Disease-Modifying Treatment (DMT), exhibit mild symptoms that do not require hospitalization [50]. In the context of severe COVID-19 and the associated cytokine storms, overactive immune system responses may affect the CNS, especially in the presence of compromised BBB integrity, a condition that often coincides with relapsing-remitting MS and predisposes patients towards neuroinflammation. The effect of coronavirus infection in disease clinical and/or radiological activity remains to be thoroughly evaluated by large-scale observation trials.

The potential of SARS-CoV-2 for direct CNS invasion poses a special challenge for MS. Coronaviruses have been previously shown to invade the CNS (reviewed in [51,52]) and to induce direct neuron loss [34]. A similar potential for SARS-CoV-2 remains to be elucidated; however, if proven, it would suggest that COVID-19 in the context of MS further predisposes PwMS towards neurodegeneration, a pathological component also recognized early in the course of the disease. This assumption is of special significance in the case of middle-aged PwMS. These individuals present more often with progressive forms of MS, compared to younger patients, indicative of the neurodegenerative process as a major source of MS-related disability for this age group [53], As middle-aged individuals are more susceptible to severe COVID-19 [6] and the peak prevalence of MS seems to be at ages 50–55 in several ethnic populations [54,55,56,57,58,59], COVID-19 is likely to pose significant implication for neurodegeneration, especially in relevance to progressive and/or active progressive forms of the disease. Moreover, middle-aged PwMS exhibit other age-related significant comorbidities, such as cardiovascular and cerebrovascular disease, hypertension, diabetes, and other autoimmune disease that are known to affect both COVID-19 and MS prognosis [60,61].

Interestingly, at least in an in vivo experimental setting, human coronaviruses exhibit a potential for long-term latency in the CNS, even a year following the recovery of acute encephalitis [62,63]. At the event that a similar potential is exhibited by SARS-CoV-2, persistence of the virus in the CNS is likely to carry implication for disease exacerbation also in PwMS who recovered from COVID-19. In this respect, the clinical implication of SARS-CoV-2 infection in PwMS needs to be carefully evaluated in long-term prospective studies that assess not only physical disability measurements but also cognition, patient-reported outcomes, and quality of life, thus aiming to elucidate COVID-19-related long-term effects on MS-related neurological status and beyond.

With respect to prophylactic DMT administered for RRMS and active-progressive MS, there is currently inadequate evidence that these treatments pose a real risk for PwMS. However, as these treatments are known to exert immunomodulatory function, their presumed effect on COVID-susceptibility and the related disease severity poses a special challenge for the MS specialists, in terms of medication management. Several Neurological Societies have published recommendations and practice guidelines for DMT management in the context of SARS-CoV-2 pandemic and these recommendations seem to add to a relative consensus. As a general rule, with the exception of PwMS exhibiting fever and/or symptoms of respiratory infection and who are recommended to temporarily interrupt DMT, no radical alterations in the treatment scheme is counseled [64]. This recommendation is particularly followed for first-line treatments, especially IFN-β and glatiramer acetate and, to a lesser extent, for oral first-line DMT dimethyl fumarate and teriflunomide, agents that are not associated with a significantly increased and/or alarming risk of infection [65]. Second-line DMT administration, especially fingolimod due to treatment-associated lymphopenia, dictate the close laboratory monitoring of hematological parameters and increased alertness towards treatment discontinuation in the event of infectious symptoms. Moreover, initiation of these DMTs during the pandemic is strongly discouraged, unless otherwise indicated by increased disease activity and a lack of alternative treatments. Moreover, treatment discontinuation has been associated with a significant rebound in the disease activity, thus posing special considerations for individuals with highly active MS. For this reason, extended interval dosing (EID) for natalizumab and other monoclonal antibodies, such as ocrelizumab, as well as careful monitoring of fingolimod-associated lymphopenia are recommended strategies in order to balance DMT efficacy and safety issues in the scope of COVID-19 [47,64]. EID also offers the advantage of reducing the patients’ exposure to the health system and the associated risk for SARS-CoV-2 infection. The initiation of cell-depleting treatments, such as the B-cell-targeting agents ocrelizumab and rituximab and the less selective cladribine and alemtuzumab, is generally counter-recommended during the pandemic [64].

Interestingly, contrary to the initial concerns, not all DMTs appear to be detrimental in case of COVID-19 and a few may even appear beneficial, at least for the management of severe COVID-19 and the associated over-activity of the immune system. In this respect, interferon-beta, exerting a previously known anti-viral effect [66] (ClinicalTrials.gov Identifier: NCT04276688) and fingolimod, due to its relative immunosuppressant effect [67] (ClinicalTrials.gov Identifier: NCT04280588), are currently under trial as potential treatments for COVID-19 in the general population. [64]. Whether these DMTs offer an advantage to PwMS in the event of concurrent COVID-19 remains to be verified by real-world data. In line with this assumption, a recent report of a PwMS treated with ocrelizumab and concomitant COVID-19 provides further evidence of a potential favorable effect [68]. In spite of efficient B-cell depletion expected by the anti-CD20 infusion, the patient upon COVID-19 additionally presented with normal leucocyte and lymphocyte counts, elevated C-reactive protein, a slight increase in interleuchin-6 (IL-6) levels, and moderate IgG hypogammaglobulinemia. Although increased IL-6 and inflammatory markers are laboratory findings previously positively associated with poor prognosis of COVID-19, the patient progressed with symptom resolution within two days, without serious complications. In this patient, it is likely that the immune-modifying effect of B-cell depletion prevented the over-activity of the T-cell immune compartment, recognized as a key mediator of cytokine storms and ARDS, thus preventing clinical deterioration.

Several lines of evidence also underline the importance of comorbid factors being effectively controlled in the context of COVID-19, such as diabetes. These observations carry implications for COVID-19 management in PwMS, a disease frequently coinciding with systemic comorbidities. Diabetes and/or uncontrolled hyperglycemia is a frequent finding in hospitalized patients with COVID-19, associated with prolonged hospitalization and poor outcomes, even increased mortality rates [69]. This report indicates the need for the careful evaluation of comorbidities in PwMS and thorough clinical and laboratory investigation upon SARS-CoV-2 infection, even in the absence of these comorbidities in the personal history.

Apart from diabetes, other systemic comorbidities of autoimmune origin may coincide with MS, and these comorbidities may be linked with demyelinating episodes exacerbated by SARS-CoV-2 infection. Reports of patients with COVID-19 exhibiting leukocytosis, thrombocytopenia, elevated prothrombin time and partial thromboplastin time, elevated levels of fibrinogen and D-dimer, as well as the presence of anticardiolipin IgA antibodies and anti-β2-glycoprotein IgA and IgG antibodies, indicate that the systemic response triggered by SARS-CoV-2 may induce laboratory findings consistent with Systemic Lupus Erythematosus (SLE) and/or anti-phospholipid syndrome, both frequent MS comorbidities [70,71]. Although these laboratory markers may be transiently elevated in patients with critical illness and infectious disease [72], the clinical implication of SARS-CoV-2 infection in patients with SLE and/or anti-phospholipid syndrome with or without MS (and the associated potential for demyelination) remains to be elucidated.

The potential association between SLE and COVID pathology is further indicated by the fact that the ACE2 gene is overexpressed in patients with SLE [73,74]. Hydroxychloroquine, an anti-malarial agent frequently prescribed in patients with SLE, was reported to exert an anti-viral effect on SARS-CoV-2 in vitro [75], as well as in a recent non-randomized open-label study [76]. Potential common immune pathways in the pathogenesis of SLE and COVID-19 may come into play, as indicated by excessive immune activation and cytokine dys-regulation in both conditions [73] Although existing evidence with respect to the beneficial effect of hydroxychloroquine in COVID-19 is conflicting [77], relevant trials are currently underway [78]. In the frame of frequent SLE and MS co-occurrence, it is of special importance to assess whether hydroxychloroquine administration for SLE poses a prophylactic effect against SARS-CoV-2 infection for patients with SLE and MS, and whether this potential effect is beneficial for long-term MS outcomes.

## 4. Conclusions

In summary, the SARS-CoV-2 pandemic calls for a thorough re-evaluation of the recommended practices for PwMS, with a special interest in DMT management and the evaluation of comorbidities. Increased alertness is warranted to accurately select PwMS, for which the need for treatment discontinuation for the prevention of SARS-CoV-2 infection outweighs the risk of disease exacerbation and activity rebound. Despite initial concerns, MS by itself and several DMTs, especially the first-line agents, do not seem to place the PwMS at a significant risk of infection. Several countries, via the cooperation of the regulating authorities with the scientific community, proceed in respective modifications in the health system services in order to reduce the exposure of PwMs to the health system and to attenuate the associated risk of infection. As the SARS-CoV-2 pandemic is expected to progress into endemic prevalence, treating physicians need to emphasize sustaining the ambitious goals of minimal or even absent disease activity and progression, posed by the several prophylactic treatment options available to date, that may be applied in an individualized (“precision medicine”) [79] approach.
